# IUTF Dataset: Enabling Cross-Border Resource for Analysing the Impact of Rainfall on Urban Transportation

**DOI:** 10.1038/s41597-025-06336-3

**Published:** 2025-11-29

**Authors:** Xuhui Lin, Qiuchen Lu, Long Chen, Jack Chin Pang Cheng, Jiayi Yan, Jingke Hong, Pengjun Zhao

**Affiliations:** 1 Phd Candidate, The Bartlett School of Sustainable Construction, University College London, London, UK; 2 Professor/Royal Academy of Engineering (RAEng)/Leverhulme Trust Research Fellow, The Bartlett School of Sustainable Construction, University College London, London, UK; 3 Assistant Professor, Department of Architecture and Civil Engineering, The City University of Hong Kong, Hong Kong, China; 4 Professor, Department of Civil and Environmental Engineering, The Hong Kong University of Science and Technology, Hong Kong, China; 5 Scientist, Department of Information Services, University of Cambridge, Cambridge, UK; 6 Professor, Chongqing University, Chongqing, China; 7 Professor, School of Urban Planning and Design, Shenzhen Graduate School, Peking University, Shenzhen, China

**Keywords:** Natural hazards, Civil engineering

## Abstract

Understanding the impact of extreme weather, particularly flooding, on urban transportation systems is critical for enhancing city resilience and traffic management. However, research and policy development are often hampered by a lack of datasets that comprehensively integrate detailed traffic dynamics, high-resolution weather information, and road network topology across multiple diverse urban environments. To address this significant gap, we present the Integrated Urban Traffic-Flood (IUTF) dataset. This open-access resource covers 40 major cities across Europe, North America, and Asia, including 21,739 sensors. The IUTF dataset uniquely combines (i) high-resolution traffic parameters derived from over 21,700 sensors (with raw data typically at 5-minute intervals, harmonised to hourly); (ii) detailed hourly precipitation data from ERA5 reanalysis, spatially aligned with (iii) the underlying road network topology for over 1 million road segments, processed from OpenStreetMap. This meticulously curated and validated dataset, created through a novel spatio-temporal harmonisation framework, enables unprecedented, cross-border analysis of weather impacts on urban mobility. It provides a foundational data resource to support applications in traffic flow prediction, infrastructure planning, and the future development of quantitative resilience models.

## Background & Summary

Urban transportation networks are fundamental to the economic and social vitality of modern cities^1^. However, these critical systems face escalating threats from extreme weather events, with flooding, in particular, emerging as a profoundly disruptive force to urban mobility in the 21st century^2^. Projections indicate a future with more frequent and intense precipitation^3^. A trend already causing global disruptions, overwhelming infrastructure, and incurring substantial economic losses—often exceeding $100 billion USD annually in recent years^4,5^, starkly illustrated by major incidents from Zhengzhou to the US East Coast and Europe^6–9^. Beyond immediate inundation, rainfall triggers complex cascading effects and non-linear interactions within transport networks^10–13^, making prediction and management exceptionally challenging without comprehensive, integrated data. This research presents a data descriptor that provides the foundational infrastructure for such analyses, with data crucial for applications ranging from vulnerability assessments and predictive modelling to the strategic planning of resilient infrastructure and adaptive traffic management. While significant public datasets for urban traffic analysis exist—such as METR-LA^14^, the PEMS family^15,16^, and the UTD19^17^—and meteorological monitoring has advanced with tools like weather radar and reanalysis products such as European Centre for Medium-Range Weather Forecasts (ECMWF) Reanalysis v5 (ERA5)^18,19^, a critical gap remains when addressing traffic-weather interactions at a detailed, multi-city scale. Specifically, the public datasets have three key limitations:

- **Lack of Integrated Traffic and Detailed Weather Data**: Most existing datasets focus on either traffic or weather, seldom providing concurrent, high-resolution data from both domains directly linked at the road network level. For example, widely used traffic datasets such as METR-LA (207 sensors in Los Angeles) and PEMS-BAY (325 sensors in San Francisco Bay Area) provide detailed traffic measurements but lack corresponding meteorological data, while weather datasets like ERA5 offer comprehensive climate information at ~31 km resolution but are not spatially aligned with transportation infrastructure. Researchers attempting to combine these sources face substantial technical barriers including data format incompatibility. coordinate system mismatches (local vs. geographic coordinate systems), and temporal alignment complexity (local time zones vs. UTC).
- **Insufficient Spatio-Temporal Alignment:** When both data types are available, they often lack the necessary spatial and temporal harmonisation (e.g., traffic sensors aligned with relevant weather data grids, consistent time zones and resolutions) required for robust interaction analysis. Spatial misalignment occurs due to scale mismatches (ERA5’s ~31 km grid resolution vs. point-based traffic sensors), coverage disparities (weather stations spaced 10–25 km apart vs. traffic sensors at 0.5–2 km intervals), and boundary effects where urban areas span multiple weather grid cells. Temporal misalignment arises from time zone complexity (traffic data in local time across multiple time zones vs. weather data in UTC), resolution inconsistencies (5-minute traffic intervals vs. hourly weather data), and measurement timing differences (accumulated weather values vs. instantaneous traffic readings).
- **Limited Multi-City Scope for Integrated Datasets**: While some regional efforts integrate traffic and weather data^20^, comprehensive datasets covering diverse international cities remain extremely rare in the public domain. Most traffic-weather interaction studies focus on single metropolitan areas, with multi-city studies typically constrained to single countries or regions, limiting cross-climatic and cross-cultural generalizability. When multi-city integration is attempted, researchers face methodological inconsistencies (different sensor technologies and data collection protocols across cities), incomplete temporal coverage (varying data availability periods), and weather data heterogeneity (combining different sources with varying spatial resolutions and quality standards).

To address these fundamental limitations, this paper introduces the Integrated Urban Traffic-Flood (IUTF) dataset, a comprehensive, open-access resource spanning 40 major cities across Europe, North America, and Asia. The IUTF incorporates three key data components: (1) high-resolution traffic measurements from 21,739 sensors recording at 5-minute intervals, yielding 411,631 temporal observations per sensor; (2) detailed road network information encompassing the topology and attributes of 1,067,085 road segments; and (3) hourly precipitation data from ERA5 reanalysis, providing 3,356 city-days of meteorological observations across all cities during the study period.

The IUTF uniquely integrates traffic measurements with spatially and temporally aligned precipitation data and road network topology from OpenStreetMap (OSM)^21^. This integration creates a novel resource for analysing weather-traffic interactions across diverse urban contexts, providing the data foundation that enables researchers to conduct comprehensive analysis of how extreme weather events impact urban mobility patterns while maintaining the temporal resolution necessary for capturing both immediate and longer-term effects of precipitation on traffic flow. The IUTF dataset advances the field through several key contributions:

- **A Large-Scale, Multi-City Integrated Resource**: It provides an unprecedented, standardised collection of traffic, weather, and network data for 40 diverse global cities, specifically designed to enable robust comparative urban resilience studies.
- **A Novel and Replicable Harmonisation Framework**: We develop and implement a systematic methodology for the spatial and temporal harmonisation of point-based traffic sensor data, linear road networks, and gridded precipitation data, offering a transferable approach for future global urban data integration.
- **Ensuring Data Quality and Accessibility**: The dataset undergoes rigorous quality control procedures, including automated consistency checks and manual validation, to ensure reliability across all cities. The data is structured in accessible formats that support diverse analytical workflows while maintaining technical rigor, facilitating both academic research and practical applications in urban transportation management.
- **Providing Data Infrastructure for Advanced Impact and Resilience Analysis**: By providing deeply integrated and contextualised data, IUTF creates the essential empirical foundation needed to facilitate sophisticated investigations into how precipitation events affect urban mobility patterns and to support the future development and validation of quantitative resilience models.

## Methods

**Fig. 1:**
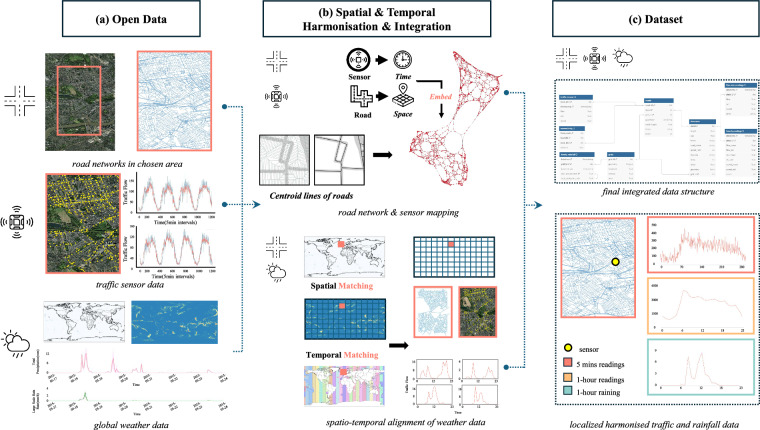
Overview of the IUTF Dataset Generation Process. (**a**) Raw data inputs include delineated urban road networks. (**b**) This stage involves processing the road network (e.g., centroid line extraction), mapping traffic sensors to road segments, and performing spatial and temporal matching of weather data with the road network and traffic data, including time zone Standardisation and resolution aggregation. (**c**) The final output is the structured IUTF dataset, featuring a clear schema for road networks, traffic readings (original 5-minute and aggregated hourly), and aligned hourly rainfall data, enabling localised and network-wide analysis of traffic-weather interactions.

Our data workflow for creating the IUTF dataset comprises three main stages: (1) sourcing, selection, and initial processing of open data for traffic flow, road networks, and precipitation; (2) comprehensive spatio-temporal harmonisation, integrating these diverse data types into coherent urban network structures; and (3) compilation of the final structured dataset, including derived analytical matrices and rigorous technical validation. We employ a consistent and standardised pipeline to process and align data from established sources like UTD19, OpenStreetMap, and ERA5 across 40 global cities. Figure 1 provides a visual overview of this workflow.

### Data collection and sources

The IUTF Dataset combines three primary data sources: high-resolution traffic measurements from UTD19, comprehensive road network data from OpenStreetMap, and precipitation records from ERA5 reanalysis. Each data source was carefully selected to ensure temporal alignment, spatial compatibility, and data quality consistency across all study cities.

#### Traffic flow data

The traffic flow measurements are sourced from UTD19, a benchmark urban traffic dataset that covers 40 global cities. For our study, we extracted data from 40 cities that demonstrated consistent sensor coverage and data quality between 2015 and 2017. The raw data includes three fundamental traffic parameters: flow (vehicles per hour), and occupancy (percentage). These measurements are collected through various types of stationary sensors, including inductive loop detectors, supersonic detectors, and cameras, with each sensor providing readings at hourly intervals. The sensor density varies across cities, ranging from 150 to 850 sensors per city, with an average spacing of approximately 500 meters along major arterials.

#### Road network information

Road network data is obtained from OpenStreetMap (OSM), accessed through the OSMNX Python package. We extracted the complete road network for each city, including attributes such as road type, speed limits, and number of lanes. The OSM data provides comprehensive coverage of the urban road network, with particularly detailed information for major roads where traffic sensors are typically located. The road network data was retrieved for the 2015–2017 period to ensure temporal consistency with the traffic sensor data and accurate sensor-to-road segment matching. The 2015–2017 timeframe was selected to maximize temporal overlap and consistent sensor coverage across all 40 cities within the UTD19 dataset, representing the optimal period that met our rigorous criteria for robust cross-city comparative analysis.

#### Precipitation data

Precipitation measurements are derived from the ERA5 reanalysis dataset, provided by the ECMWF. ERA5 offers global coverage at approximately 31-kilometer spatial resolution and hourly temporal resolution. For each city, we extracted precipitation data for the entire study period (2015–2017), including both total precipitation and precipitation rate. The ERA5 dataset was chosen for its consistent methodology across all study regions and its proven reliability in urban meteorological applications. While the spatial resolution is coarser than some local weather station networks, it provides uniform coverage and methodology across all study cities, enabling consistent cross-city analysis.

### Spatial harmonisation process

**Fig. 2:**
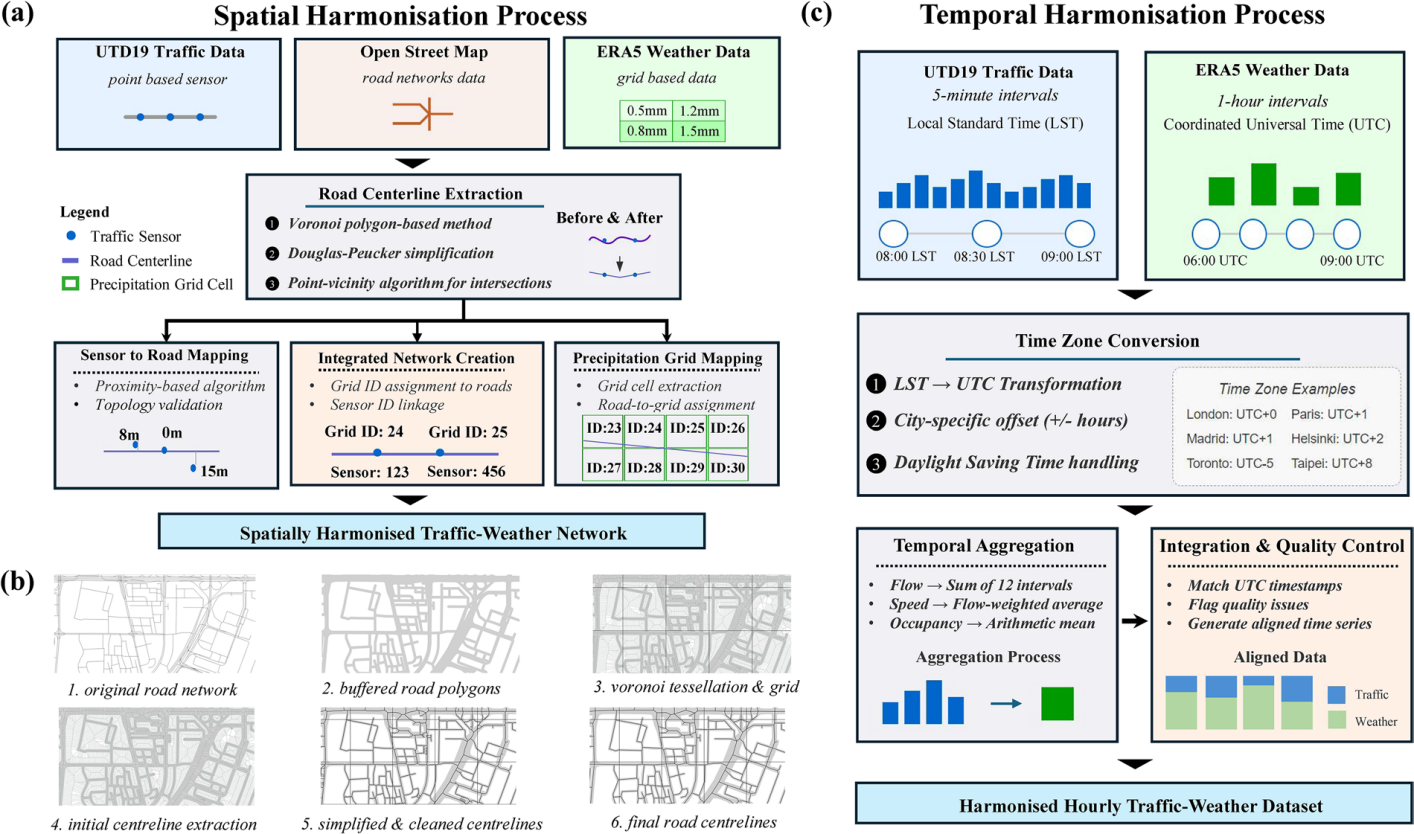
Methodology for spatial and temporal harmonisation in IUTF dataset construction. (**a**) Spatial harmonisation workflow integrating three data sources: UTD19 traffic sensors (point-based), OpenStreetMap road networks (complex geometries), and ERA5 precipitation data (grid-based). The process includes sensor-to-road mapping, centreline extraction using Voronoi algorithms, and precipitation grid alignment to create an integrated network with linked sensor and precipitation identifiers. (**b**) Road centreline extraction process using Voronoi polygon-based method, progressing from original road networks to final simplified centrelines. (**c**) Temporal harmonisation process converting traffic data from lo LST to UTC, handling time zone differences and daylight-saving time, followed by temporal aggregation of 5-minute traffic data to hourly intervals to align with precipitation data.

The integration of traffic sensor data, road networks, and precipitation measurements required a comprehensive spatial harmonisation approach to ensure consistent geographical representation and analysis capabilities. As illustrated in Fig. 2(a), our methodology was designed to ensure consistent geographical representation and robust analysis capabilities by addressing three key spatial integration challenges: the processing and simplification of road networks, precise mapping of traffic sensor locations to these networks, and accurate alignment of gridded precipitation data with the road infrastructure.

#### Road network processing and sensor integration

The spatial harmonisation of traffic sensor data with road networks presents a fundamental challenge in multi-source transportation data integration. Raw OpenStreetMap road networks contain complex geometries with varying levels of detail, while traffic sensors from UTD19 are provided as discrete point locations without explicit road segment associations. This stage addresses two critical requirements (as shown in Fig. 2(a)): first, transforming complex OSM road geometries into simplified but topologically accurate centreline representations suitable for network analysis; and second, establishing precise spatial relationships between point-based sensor locations and their corresponding road segments. This process is essential for creating a unified spatial framework that enables subsequent integration with gridded precipitation data.

##### Centreline network extraction

**Fig. 3:**
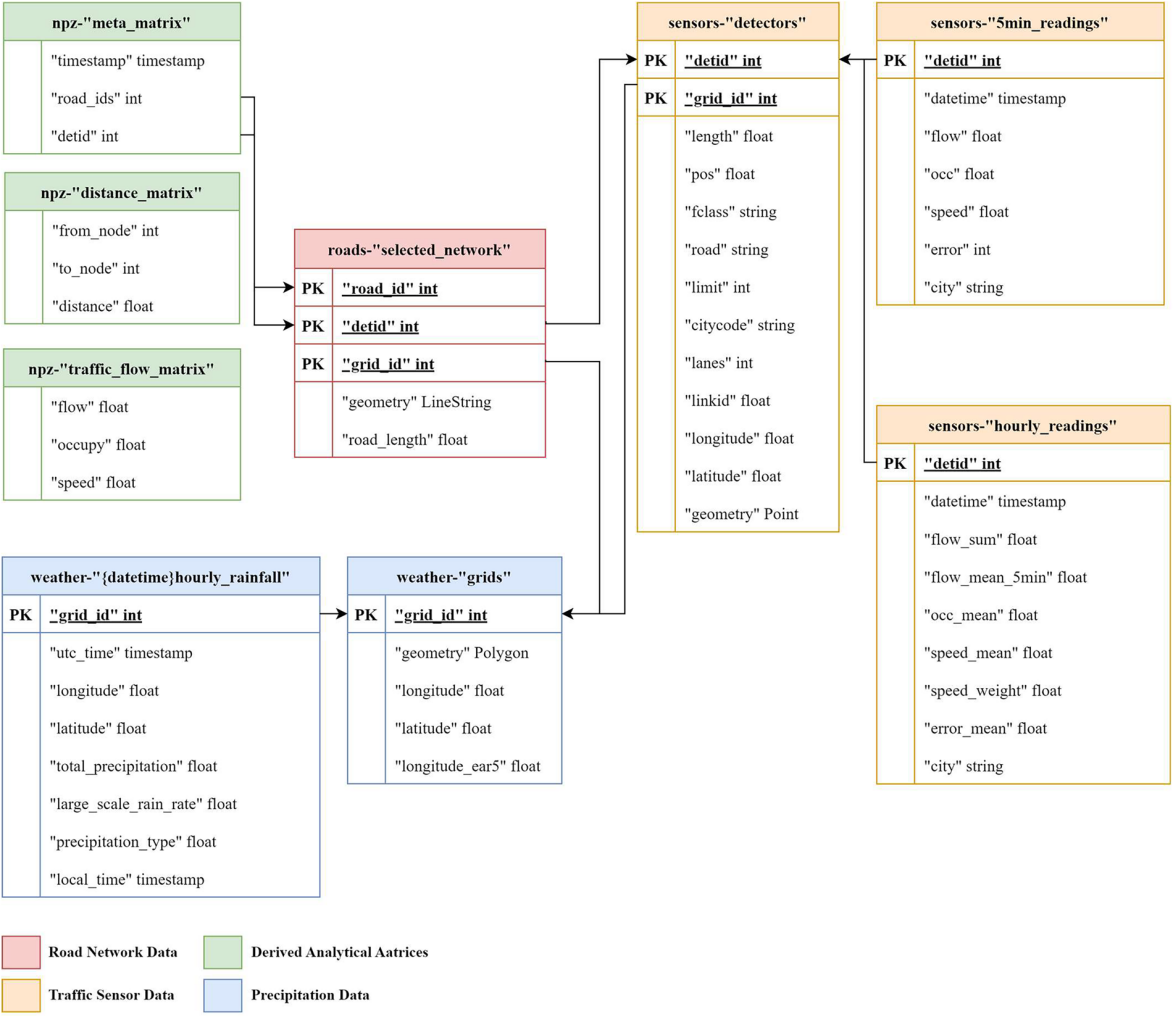
Relational Schema of the IUTF Dataset. This diagram illustrates the organisation of the IUTF dataset, color-coded by primary data category: Road Network Data (red), Traffic Sensor Data (orange), Precipitation Data (blue), and Derived Analytical Matrices (green). Key datasets such as roads, detectors, five_min_readings, hourly_readings, and hourly_rainfall are shown with their primary fields and interconnections, facilitated by identifiers like road_id, detid, and grid_id.

**Fig. 4:**
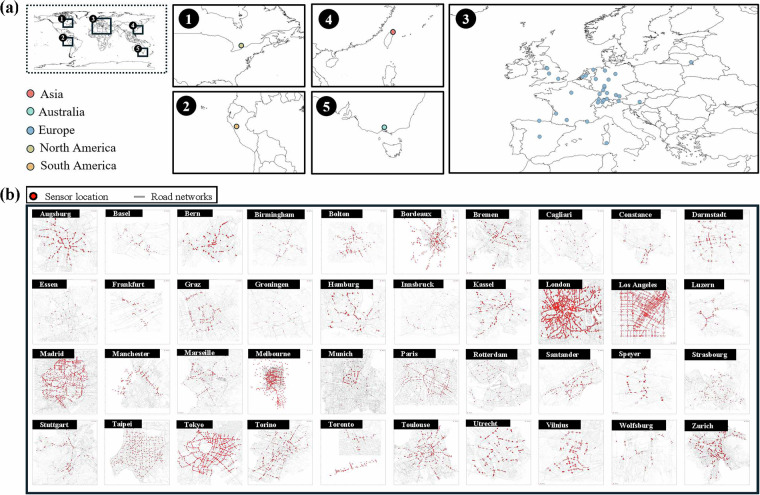
Overview of Selected Cities in the IUTF Dataset, Showing Geographical Locations and Individual Road Network with Sensor Placements. (**a**) global map indicating the locations of the 40 selected cities, color-coded by continent (Asia, Australia, Europe, North America, South America), with inset maps providing regional zoom-ins for North America (1), South America (2), Europe (3), East Asia (4), and Oceania (5). (**b**) spatial visualisations of the road network structure (grey lines) and traffic sensor locations (red dots) for each of the 40 cities, illustrating the varying network typologies and sensor coverage densities across the dataset.

**Fig. 5:**
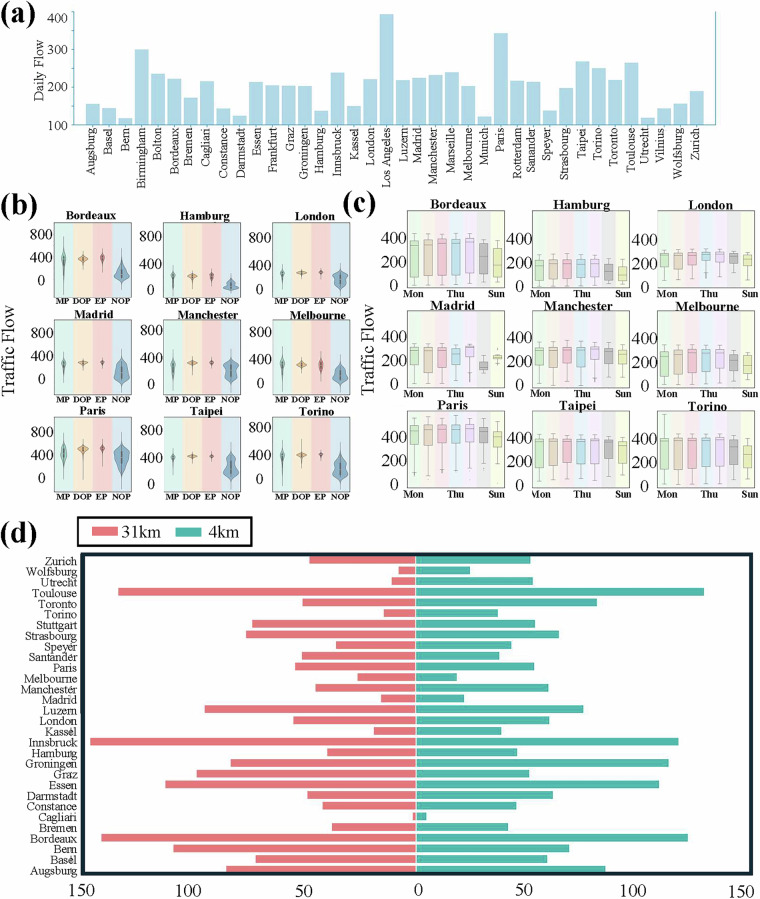
Validation of Traffic Flow Patterns and ERA5 Precipitation Data Consistency in the IUTF Dataset. (**a**) Average Daily Traffic Flow: Bar chart illustrating the average daily traffic flow across all included cities, providing a baseline understanding of typical traffic volumes. (**b**) Traffic Flow by Time of Day: Violin plots for selected cities (e.g., Bordeaux, Hamburg, London, Manchester, Melbourne, Paris, Taipei, Torino) showing the distribution of traffic flow across different time periods (MP, DOP, EP, NOP stands for Morning Peak, Daytime Off-Peak, Evening Peak, Night Off-Peak), highlighting diurnal patterns. (**c**) Weekly Traffic Flow Distribution: Box plots for the same selected cities depicting the distribution of traffic flow across days of the week (Monday to Sunday), illustrating weekly traffic rhythms. (**d**) Precipitation Data Comparison: Tornado plot comparing the distribution or frequency of precipitation events as captured by the ERA5 (31 km resolution, hourly) data used in IUTF (pink bars) against a higher-resolution dataset (4 km resolution, daily) for each city (or a representative subset).

**Fig. 6:**
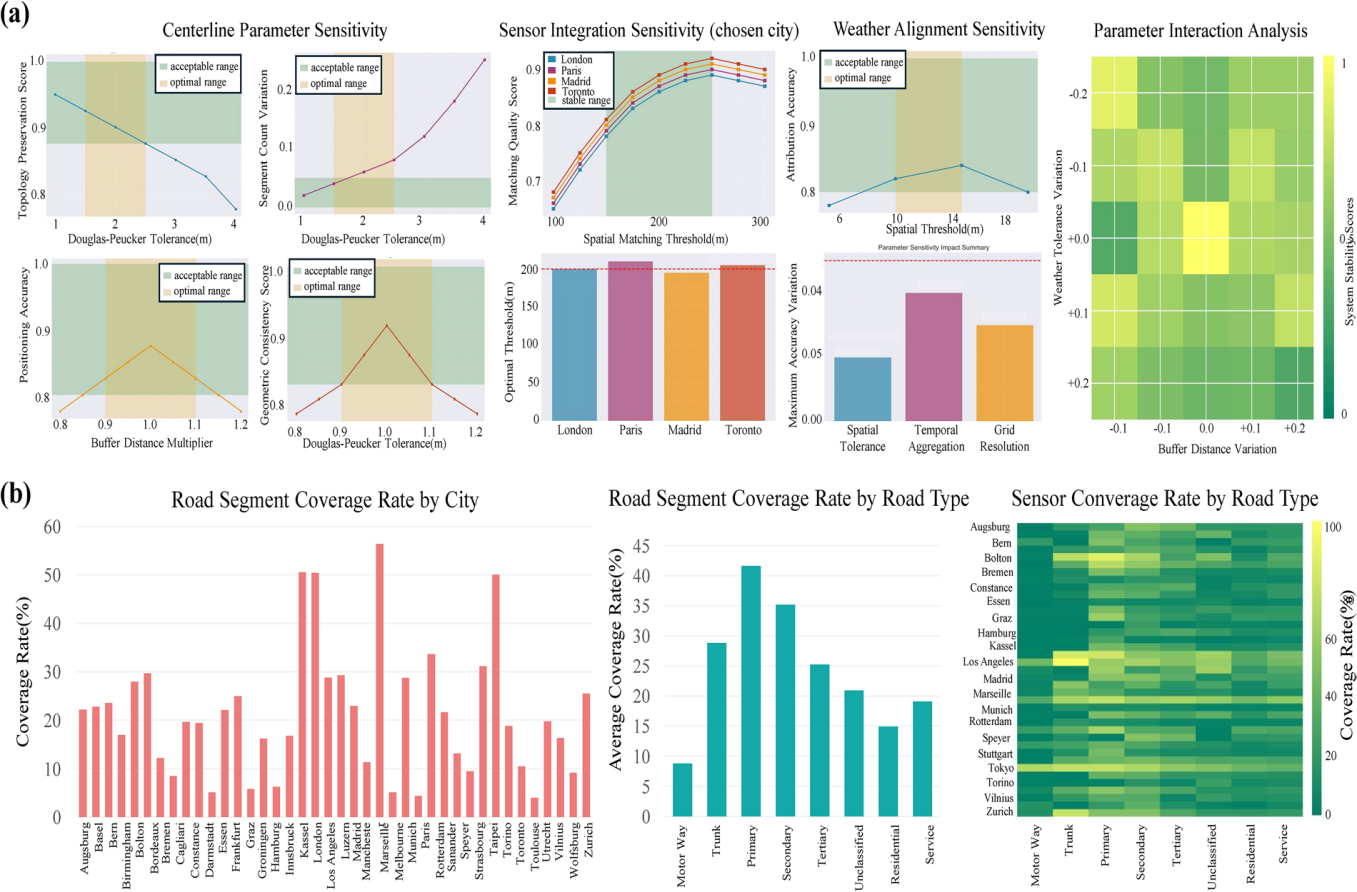
Parameter Sensitivity Analysis and Spatial Coverage Assessment of the IUTF Dataset Processing Pipeline. Parameter sensitivity analysis and spatial coverage characteristics of the IUTF dataset. (**a**) Comprehensive parameter sensitivity analysis showing robustness across four critical processing components: centreline parameter sensitivity (Douglas-Peucker tolerance and segment count variation), sensor integration sensitivity for selected cities (spatial matching threshold performance), weather alignment sensitivity (spatial tolerance and temporal aggregation impacts), and parameter interaction analysis (system stability scores under combined parameter variations). Green shaded areas indicate acceptable parameter ranges, while orange areas represent optimal ranges. (**b**) Spatial coverage assessment showing road segment coverage rates by city (left), average coverage rates by road functional classification (middle), and sensor coverage heatmap by road type across all cities (right).

The first stage, Centreline Network Extraction, is visually detailed in the six-panel workflow shown in Fig. 2(b). The process begins with the original road network from OSM (Fig. 2(b)-1), which often contains complex multi-lane geometries, detailed intersection configurations, and varying levels of geometric detail across different road types. These roads are then converted into buffered polygons (Fig. 2(b)-2) using appropriate buffer distances that account for road width characteristics when available in OSM attributes, or standard geometric assumptions based on road functional classification when width information is absent. Following this, Voronoi tessellation^22^ is applied to the buffered polygons (Fig. 2(b)-3) to derive the medial axis through computational geometry algorithms, where the resulting Voronoi cell boundaries represent the geometric centreline of each road corridor (Fig. 2(b)-4). This raw centreline geometry is subsequently refined using the Douglas-Peucker algorithm^23^ to create simplified and cleaned centrelines (Fig. 2(b)-5), which systematically removes redundant vertices while preserving essential geometric characteristics and maintaining critical topological relationships. The final step involves using a point-vicinity algorithm to refine intersection topology (Figs. 2(b)-6), ensuring proper connectivity between road segments and resolving issues such as overshooting or undershooting lines at network nodes. This comprehensive approach transforms complex, multi-lane geometries into representative single-line centrelines, a representation that is critical for integrating sensor data reflecting aggregate flow conditions across multiple lanes.

##### Sensor-to-road segment matching

Once the topologically sound centreline network is established, traffic sensors (sourced from UTD19 as point locations) are integrated through the Sensor-to-Road Segment Matching stage, as depicted in the overall workflow (Fig. 2(a)). We implement a proximity-based matching algorithm that employs nearest-neighbour analysis to identify the most suitable road segment for each sensor location. The algorithm operates by calculating the geometric distance between each sensor and candidate road segments within its vicinity, specifically measuring the shortest perpendicular distance from the sensor point to each road segment centreline rather than using arbitrary endpoint distances.

##### Topological validation of matches

The initial automated matches are then subjected to rigorous Topological Validation of Matches, a critical quality control step that addresses the limitations of purely distance-based matching approaches. This validation process systematically examines each sensor-road association to identify and resolve potential ambiguities, particularly in complex geometric scenarios such as multi-lane highways where sensors may be equidistant from multiple roadway facilities, intersections where sensor positioning relative to approach/departure configurations affects appropriate assignment, and closely parallel roads where functional classification and directional analysis are required to determine correct associations.

#### Precipitation data integration

The integration of meteorological data with transportation networks presents a significant methodological challenge due to fundamental differences in spatial representation and resolution between these data sources. ERA5 reanalysis provides precipitation data at approximately 31 km spatial resolution in a regular grid format, while road networks consist of linear features with highly variable spatial density and geometric complexity. This spatial scale mismatch creates substantial difficulties for establishing meaningful relationships between weather conditions and traffic patterns, as a single ERA5 grid cell may encompass multiple distinct road segments with potentially different precipitation exposure characteristics. Thus, this stage addresses the fundamental challenge of accurately aligning gridded meteorological data with fine-scale transportation infrastructure.

##### Spatial overlay and grid-road intersection analysis

The first stage, Spatial Overlay and Grid-Road Intersection Analysis, begins by extracting only the relevant ERA5 grid cells that geometrically intersect with each city’s road network boundary, creating a focused precipitation dataset for each urban area while significantly reducing computational demands. The process then employs computational geometry algorithms to determine precise intersection relationships between linear road segments and rectangular grid cells. This analysis requires coordinate system harmonisation, as road networks typically use local projected coordinate systems while ERA5 data employs WGS84 geographic coordinates, necessitating appropriate datum transformations to maintain spatial accuracy. For each road segment, the algorithm calculates the exact geometric relationship with intersecting grid cells, including the proportional length of road segment within each grid cell and the total intersection area, forming the foundation for subsequent precipitation attribution calculations.

#### Dual attribution methodology implementation

The second stage implements a dual attribution methodology specifically designed to support different analytical requirements through parallel processing pathways. The detailed attribution pathway preserves individual precipitation time series for each grid cell that intersects with a road segment, maintaining these as indexed data structures that retain the full spatial granularity of ERA5 data and enable analysis of precipitation variability along extended road segments. Simultaneously, the aggregated attribution pathway creates single precipitation values for each road segment through area-weighted averaging, calculated using the formula:

1 $${P}_{{segment}}=\frac{\mathop{\sum }\limits_{i=1}^{n}{P}_{{gri}{d}_{i}}\times {L}_{i}}{\mathop{\sum }\limits_{i=1}^{n}{L}_{i}}$$

where ${P}_{{segment}}$ represents the final precipitation value for the road segment, ${P}_{{gri}{d}_{i}}$ is the precipitation value in grid cell $i$, ${L}_{i}$ is the length of road segment within grid cell $i$, and $n$ is the total number of grid cells that intersect with the road segment. This weighted averaging approach ensures that grid cells containing longer portions of a road segment contribute proportionally more to the final precipitation attribution, accounting for the varying spatial influence of different grid cells on individual road segments.

##### Technical implementation and computational optimization

To handle the computational demands of processing extensive road networks against precipitation grids, the implementation employs several optimisation strategies. Spatial indexing structures, specifically R-tree algorithms, are used to efficiently identify potential grid-road intersections and reduce computational overhead while maintaining complete accuracy in intersection detection. The algorithm also employs minimum bounding rectangle calculations to pre-filter grid cells that cannot possibly intersect with road segments, reducing processing time by eliminating unnecessary geometric calculations. For road segments that span multiple cities or extend across large geographic areas, the processing is partitioned spatially to enable parallel computation and manage memory requirements effectively.

#### Temporal harmonisation process

The temporal harmonisation process addresses the challenge of aligning traffic measurements collected at 5-minute intervals in Local Standard Time with precipitation data provided at hourly intervals in Coordinated Universal Time. The objective is to create temporally synchronised datasets that enable precise analysis of cause-effect relationships between precipitation events and traffic responses while maintaining the temporal resolution necessary for capturing both immediate and longer-term weather impacts.

##### Time zone standardisation

The temporal alignment of multi-source urban data presents fundamental challenges when datasets originate from different temporal reference systems and measurement frequencies. Global urban traffic datasets inherently use local time standards that reflect regional timekeeping practices and local traffic patterns, while meteorological reanalysis products typically employ standardised universal time coordinates to ensure consistency across global coverage areas. This temporal heterogeneity creates significant complications for integrated analysis, as the same physical time moment may be represented by different timestamp values across datasets, and seasonal time adjustments (daylight saving time) introduce additional temporal discontinuities that can substantially affect traffic pattern interpretation. Furthermore, the different measurement intervals -5-minute traffic observations versus hourly precipitation data - require systematic temporal alignment to enable meaningful weather-traffic interaction analysis. The complexity increases when considering 40 globally distributed cities spanning multiple time zones with varying daylight-saving time policies and historical time zone changes during the study period (2015–2017). This stage (as shown in Fig. 2(c)) addresses the critical requirement of establishing a unified temporal framework that enables accurate synchronisation of traffic and weather observations while preserving the temporal integrity of both datasets.

##### Time zone mapping and DST handling

To address the temporal misalignment, all traffic measurements were converted from their native LST to UTC using a systematic conversion algorithm. This process involved establishing a comprehensive time zone mapping for all 40 cities, accounting for city-specific UTC offsets (examples of which are provided in Fig. 2(c)) and meticulously handling daylight-saving time (DST) transitions where applicable. The conversion algorithm specifically addresses DST transitions through systematic procedures that manage “spring forward” periods where local time jumps from 2:00 AM to 3:00 AM, and “fall back” periods where 1:00–2:00 AM occurs twice in local time. These procedures involve examining timestamp context and applying disambiguation rules based on surrounding data patterns.

##### UTC conversion implementation

The conversion follows the systematic formula:

2 $${UT}{C}_{{time}}={LS}{T}_{{time}}-{Offse}{t}_{{UTC}}-{DS}{T}_{{adjustment}}$$

where ${LS}{T}_{{time}}$ represents the original local standard time timestamp, ${Offse}{t}_{{UTC}}$ is the city-specific UTC offset, and ${DS}{T}_{{adjustment}}$ represents the additional offset (typically 1 hour) applied during daylight saving periods. A comprehensive time zone reference table documenting standard UTC offsets and DST schedules for each city during the study period (2015–2017) informed this conversion algorithm, ensuring direct temporal linkage with the UTC-based ERA5 precipitation data. The process includes validation procedures to ensure temporal consistency and identify potential conversion errors through timestamp sequence analysis and traffic pattern verification.

#### Temporal resolution aggregation

The temporal aggregation of high-frequency traffic measurements presents significant methodological challenges due to the need to preserve different statistical characteristics of various traffic parameters while achieving temporal alignment with meteorological data. Traffic flow, speed, and occupancy measurements each represent fundamentally different physical phenomena with distinct statistical properties and temporal variability patterns that require parameter-specific aggregation approaches to maintain their analytical significance. Simple temporal averaging may obscure critical traffic dynamics, particularly during transitional periods such as rush hours when traffic conditions change rapidly within short time intervals. Thus, this stage (Fig. 2(c)) addresses the fundamental requirement of transforming high-resolution temporal traffic data into hourly aggregations that maintain the essential characteristics of traffic dynamics while enabling direct comparison with meteorological observations.

##### Parameter-specific aggregation methodologies

The aggregation process implements distinct approaches tailored to each traffic parameter’s characteristics. For traffic flow, originally measured in vehicles per five minutes, an hourly total is derived using direct summation:

3 $${Flo}{w}_{{hourly}}=\mathop{\sum }\limits_{j=1}^{12}{Flo}{w}_{5{mi}{n}_{j}}$$

where $j$ represents the $j$-th 5-minute interval within each hour. This approach provides a comprehensive measure of traffic intensity that accurately represents total vehicle throughput. Speed data requires a flow-weighted average of the 5-minute readings to ensure that periods with higher traffic volumes contribute more significantly to the aggregated speed:

4 $${Spee}{d}_{{hourly}}=\frac{\mathop{\sum }\limits_{j=1}^{12}\left({Spee}{d}_{5{mi}{n}_{j}}\times {Flo}{w}_{5{mi}{n}_{j}}\right)}{\mathop{\sum }\limits_{j=1}^{12}{Flo}{w}_{5{mi}{n}_{j}}}$$

This method offers a more representative value of actual traffic conditions, especially during dynamic periods like rush hours. For road occupancy, which indicates the percentage of time a sensor detects vehicle presence, hourly values are computed as the arithmetic mean:

5 $${Occupanc}{y}_{{hourly}}=\frac{1}{12}\mathop{\sum }\limits_{j=1}^{12}{Occupanc}{y}_{5{mi}{n}_{j}}$$

yielding a stable indicator of road utilisation by smoothing out short-term fluctuations less critical for weather-impact analysis.

## Data Records

The IUTF Dataset, designed for analysing the complex interactions between urban mobility and weather phenomena^24^ (10.6084/m9.figshare.30022807.v1). This resource covers 40 cities across Europe, North America, and Asia, with temporal coverage primarily spanning 2015–2017. Collectively, the dataset encompasses approximately 400,000 road segments, measurements from around 23,627 traffic sensors (including original 5-minute readings and hourly aggregations), and data from about 140 precipitation grid cells aligned with the ERA5 reanalysis product. Each sensor provides 411,631 temporal observations over the study period, creating a comprehensive resource with 3,356 city-days of meteorological observations across all cities. The total uncompressed dataset volume is 1.61 GB, stored in highly efficient formats to optimize both storage and computational performance. Each city’s data is organised into a consistent folder structure that facilitates systematic navigation and analysis across different urban contexts, with data for individual cities ranging from 150 to 850 sensors per city depending on urban area size and monitoring infrastructure density.

### File formats and structure

To ensure optimal computational efficiency and broad compatibility with analytical workflows, the dataset employs two primary storage formats. Tabular and geometric data are stored using the Apache Parquet format, which provides excellent compression ratios and fast query performance for large datasets. Numerical matrices are provided in the NumPy NPZ format, enabling efficient loading and manipulation in scientific computing environments. Each city follows an identical organisational structure with clearly labelled subdirectories for different data components, ensuring consistent access patterns across all urban areas in the dataset.

### Primary data collections and variable descriptions

The IUTF dataset is structured around four interconnected primary components, as illustrated in the relational schema (Fig. 3), the IUTF dataset is structured around four interconnected primary components: road network data, traffic sensor data, precipitation data, and derived analytical matrices that facilitate advanced spatio-temporal analysis.

#### Road network data

This collection provides detailed topological representations of each city’s transportation infrastructure, including files that describe individual road segment attributes such as spatial geometry, length, functional classification, and lane counts. Network connectivity information specifies origin and destination relationships between road segments, enabling comprehensive network-based analyses.

#### Traffic sensor data

This collection offers comprehensive insights into urban traffic dynamics through sensor metadata files that detail physical characteristics, geographical locations, and associations with specific road segments and weather grid cells. Traffic measurements are provided in two temporal resolutions: original high-resolution 5-minute interval measurements for detailed temporal analysis and aggregated hourly statistics that align directly with precipitation data for integrated weather-traffic studies.

#### Precipitation data

This collection contains hourly meteorological information derived from ERA5 reanalysis and spatially aligned with road networks. Time-stamped records for each relevant weather grid cell include key parameters such as total precipitation accumulation and precipitation rates. Accompanying metadata files define the spatial geometry and geographic coordinates of weather grid cells.

#### Derived analytical matrices

To support advanced spatial-temporal modeling and network analyses, this collection provides pre-computed analytical structures including network distance matrices between road segments and multi-dimensional arrays containing aligned traffic measurements over time, structured for direct use in common analytical software environments.

## Technical Validation

To rigorously establish the integrity, internal consistency, and overall fitness of the IUTF dataset for its intended purpose—the detailed analysis of urban traffic dynamics under the influence of precipitation events—a multi-faceted validation strategy was employed. This strategy provides comprehensive evidence of the dataset’s quality and utility. The validation, detailed in the subsequent sections, proceeds by first examining the foundational aspects of the dataset, including its extensive geographical and network coverage, the baseline characteristics of its core traffic data, and the suitability of the chosen precipitation data source (Section 4.1). Following this, we demonstrate the dataset’s sensitivity and analytical power in detecting and quantifying traffic responses to rainfall, including the general impacts of precipitation, dynamic peri-event changes, and discernible dose-response relationships with varying rainfall intensities (Section 4.2). Finally, the validation illustrates the dataset’s capacity to uncover more complex phenomena, such as differentiated macroscopic responses within road networks and statistically robust, aggregated patterns of traffic disruption across diverse urban contexts (Section 4.3). These analyses collectively substantiate the IUTF dataset as a robust resource for advancing research in urban mobility and climate resilience.

### Validation of dataset coverage, baseline characteristics, and source data integrity

#### Geographical scope and network diversity

A foundational aspect of the IUTF dataset’s utility lies in its extensive geographical scope and the inherent diversity of the urban environments it represents. Figure 4 provides a visual testament to this, showcasing the global distribution of the 40 selected cities across multiple continents and offering thumbnail visualisations of each city’s unique road network topology and traffic sensor layout. This diverse representation, encompassing a variety of network structures and sensor densities, establishes the dataset’s strong potential for broad-ranging comparative studies on urban mobility and resilience. Beyond the spatial coverage, the integrity of the core data components was assessed. The traffic data, a critical input, exhibits plausible and expected temporal patterns, as illustrated in Fig. 5 (a–c). Figure 5(a) displays the average daily traffic flow across the cities, providing a baseline of traffic volume. Further characterisation, shown in Fig. 5(b,c) for selected cities, reveals distinct diurnal rhythms, with clear peak and off-peak periods, as well as typical weekly variations in traffic flow. The consistent observation of these fundamental traffic patterns across the dataset substantiates the reliability and fundamental integrity of the traffic flow measurements integrated into IUTF. Given the dataset’s focus on weather impacts, the suitability of the chosen precipitation data source, ERA5 reanalysis (approximately 31 km spatial resolution), was also critically evaluated. Figure 5(d) presents a comparative analysis between the ERA5 data (31 km, pink bars) and the TerraClimate dataset^25^ (4 km, monthly, teal bars), which serves as a higher-resolution reference. The TerraClimate dataset is derived from WorldClim’s high-resolution climate normal, combined with the temporal variability of CRU Ts4.0 and JRA-55, and generated through climate-aided interpolation, making it capable of accurately reflecting regional-scale precipitation variability. The results of this comparison demonstrate a notable consistency between the two sources, with most cities showing comparable monthly precipitation estimates detected by both datasets. For instance, cities like London, Hamburg, exhibit very similar levels. While some variations exist, which can be expected due to differences in spatial resolution and detection thresholds, the overall alignment in the timing and magnitude of precipitation events suggests that, despite its coarser native resolution, the ERA5 data effectively captures the occurrence of precipitation conditions pertinent to urban-scale traffic impact analyses. This finding validates its use as the primary meteorological input for the IUTF dataset.

#### Parameter sensitivity and processing pipeline robustness

With the foundational integrity of the dataset’s scope and core components established, we next conducted a comprehensive sensitivity analysis to evaluate the robustness of the data processing pipeline itself. Our framework systematically evaluated four critical parameter groups governing the spatio-temporal harmonisation process across ± 30% ranges from baseline values, validated across all 40 cities. The centreline network construction demonstrates exceptional stability, with the Douglas-Peucker simplification tolerance achieving optimal performance at 2.0 m (topology preservation > 0.90, optimal range 1.5–2.5 m) and Voronoi buffering maintaining geometric consistency > 0.90 around the 15 m baseline with < 3% positioning accuracy impact. Similarly, traffic sensor integration exhibits robust performance with the 200 m spatial matching threshold achieving optimal coverage-quality balance across the 150–250 m range, showing cross-city consistency (coefficient of variation < 0.15) regardless of urban morphology. Furthermore, weather data alignment maintains high attribution accuracy (robustness score 0.91) at 12 m spatial tolerance with < 2% variation across the 10–15 m range, while temporal aggregation preserves correlation coefficients > 0.85 for the 5-minute to 1-hour method. Most importantly, cross-parameter interaction analysis reveals primarily additive effects with maximum combined magnitudes < 0.10, maintaining system stability > 0.80 when variations remain within ± 20% of baseline values. This comprehensive analysis establishes that our processing pipeline is robust to reasonable parameter variations across diverse urban contexts, providing evidence-based guidelines that validate the methodological foundation for subsequent validation of the dataset’s analytical capabilities in detecting traffic responses to precipitation events.

#### Spatial coverage assessment and network representativeness

Following the validation of our data processing pipeline’s robustness, we then performed a granular analysis to assess the spatial coverage and representativeness of the final, generated sensor network within each city, as illustrated in Fig. 6. This analysis provides essential context for understanding the dataset’s spatial characteristics and representativeness across diverse urban environments. The sensor-to-road mapping process successfully associated traffic sensors reach an average coverage rate of 22.1%. Coverage rates demonstrate substantial variation between cities, ranging from 4.0% in cities with limited sensor infrastructure to 56.4% in London. This variation reflects the diverse nature of transportation monitoring systems across different urban contexts, institutional frameworks, and infrastructure development priorities. The coverage distribution exhibits systematic patterns related to road functional classification, as demonstrated in Fig. 6. Trunk roads achieve the highest average coverage at 41.6%, followed by primary roads at 35.2%, secondary roads at 25.2%, tertiary roads at 20.9%, and local roads ranging from 14.9% to 19.1%. This hierarchical pattern emerges consistently across the diverse set of global cities, with the heatmap analysis revealing that most cities achieve coverage rates of 60–90% on trunk roads, declining systematically through the road hierarchy to 20–40% coverage on local facilities. The spatial distribution of sensor coverage concentrates along primary transportation corridors rather than being randomly distributed across urban networks. This concentration creates connected monitoring networks that span major arterials and highway facilities within each city. When weighted by typical traffic volume patterns associated with different road classifications, the effective coverage increases substantially above the geometric coverage rates, as higher-classification roads typically carry disproportionate shares of total urban traffic volume. This coverage assessment reveals the systematic nature of sensor deployment across diverse urban transportation networks while establishing the spatial foundation for subsequent analyses of traffic-weather interactions throughout the validation framework. The consistent hierarchical coverage patterns across cities provide confidence that observed traffic responses to weather conditions reflect systematic relationships rather than artifacts of particular monitoring configurations.

### Validation of sensitivity to rainfall impacts and quantification of effects

**Fig. 7:**
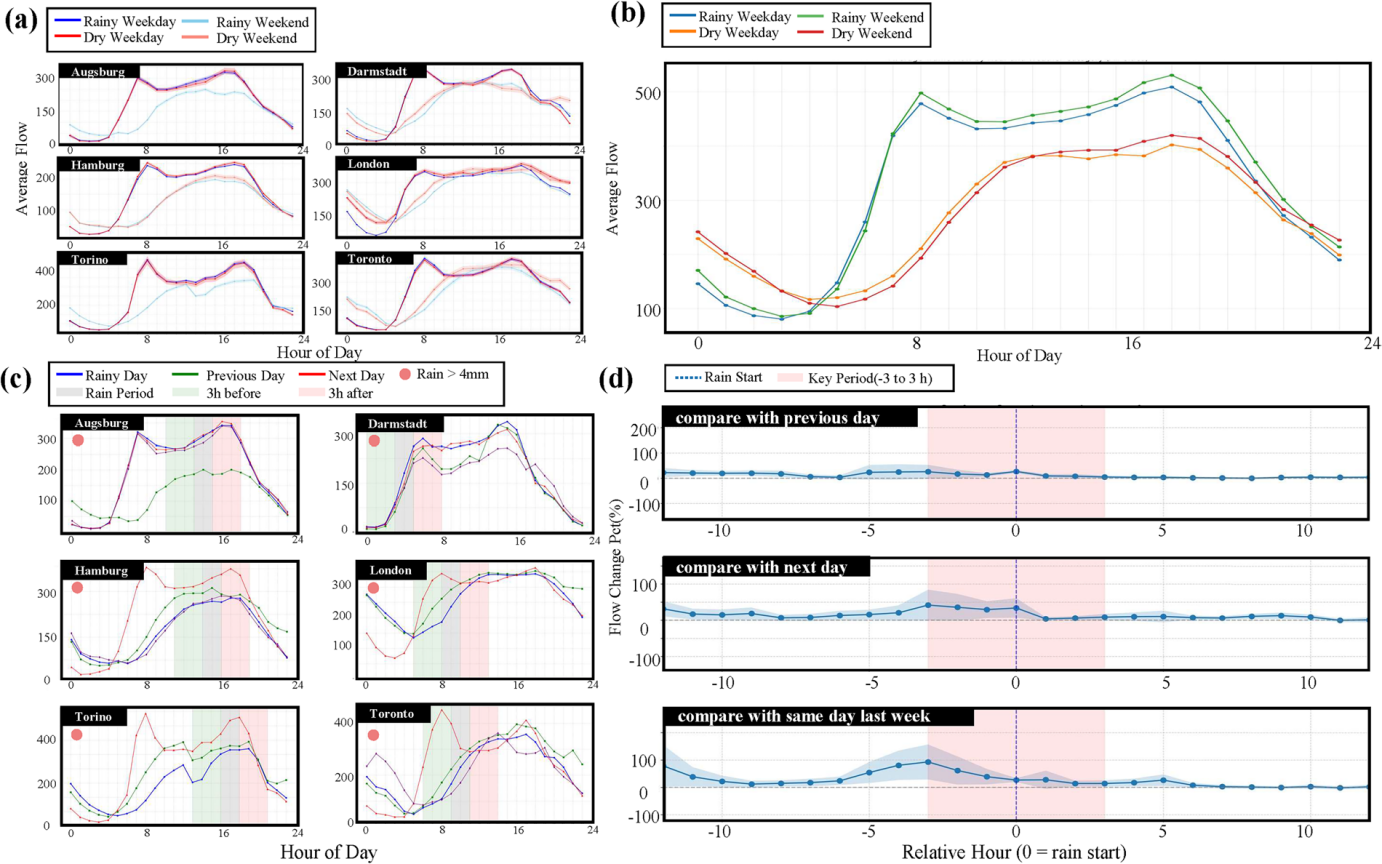
Characterising the influence of rainfall on urban traffic flow dynamics. (**a**) City-specific diurnal traffic patterns: Traffic flow profiles for selected cities comparing rainy versus dry conditions across weekdays and weekends, demonstrating city-specific variations in how precipitation affects typical daily traffic patterns. (**b**) Aggregated diurnal patterns: Average hourly traffic flow across all cities categorised by weather conditions and day type, showing dataset-wide patterns of rainfall impact on traffic throughout the day. (**c**) City-specific rainfall event analysis: Traffic flow patterns for selected cities during individual precipitation events, with shaded periods indicating rainfall occurrence and different lines showing baseline comparisons. (**d**) Aggregated rainfall event analysis: Percentage change in traffic flow relative to rainfall onset (hour 0) across all cities, using three comparison baselines (next day, previous day, and same day of previous week). The shaded area highlights the peri-rainfall period (−3 to +3 hours), demonstrating the dataset’s capability to capture statistically significant anticipatory and reactive changes in traffic flow due to rainfall events.

The following analyses are presented as a crucial technical validation of the IUTF dataset. The primary goal is to demonstrate the dataset’s sensitivity and analytical capability in detecting and quantifying the impacts of rainfall on traffic dynamics. By confirming that the data can reveal clear, dose-response relationships and other complex patterns, we validate its utility as a reliable, foundational resource for future, more in-depth resilience assessments. Having established the dataset’s scope and the integrity of its core components, the validation proceeded to assess its capability to detect and quantify the impacts of rainfall on urban traffic flow. To validate the IUTF dataset’s utility for future resilience assessments, it is crucial to first demonstrate its sensitivity in detecting traffic responses to rainfall. As shown in Fig. 7, the dataset allows for direct and robust comparisons of traffic patterns under varying weather conditions. Distinct differences emerge in diurnal traffic profiles when comparing rainy versus dry conditions, both in specific cities (Fig. 7(a)) and when aggregated across the entire dataset (Fig. 7(b)). This validates the dataset’s fundamental sensitivity in capturing the general disruptive effect of rainfall on established urban traffic patterns, a necessary first step for any subsequent resilience analysis. Furthermore, the dataset’s temporal granularity enables a detailed examination of traffic dynamics around rainfall events (Fig. 7(c),(d)). The ability to capture such fine-grained peri-event dynamics is critical, as it provides the raw empirical data needed for future studies to calculate specific resilience metrics like system recovery times and absorption capacity. This underscores the dataset’s direct applicability as a foundational resource for quantitative resilience research.

**Fig. 8:**
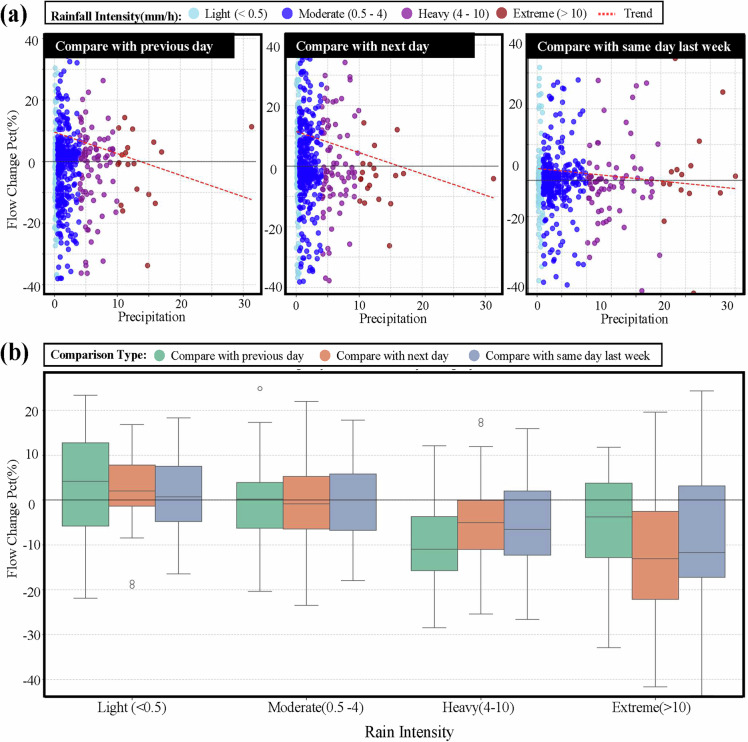
Relationship Between Rainfall Intensity and Traffic Flow Changes with Different Comparison Baselines. (**a**) Scatter plots showing traffic flow percentage change versus rainfall intensity for three comparison baselines (previous day, next day, and same day of previous week), with points coloured by rainfall intensity categories (Light < 0.5, Moderate 0.5–4, Heavy 4–10, Extreme > 10 mm/hr, defined by Met Office^26^). Trend lines indicate the general relationship between rainfall intensity and traffic impact. (**b**) Box plots summarising traffic flow changes by rainfall intensity category after outlier removal (beyond 2.0 IQR), demonstrating that increasing rainfall intensity correlates with more significant traffic flow changes.

A key requirement for any resilience-enabling dataset is the ability to support investigations into dose-response relationships. The IUTF dataset was validated for this purpose, as shown in the analysis of how traffic flow changes correlate with varying rainfall intensities (Fig. 8). Both individual event responses (Fig. 8(a)) and summarised distributions (Fig. 8(b)) consistently indicate that increasing rainfall intensity is associated with more pronounced traffic flow changes. This validation confirms that the dataset can facilitate nuanced investigations into the quantitative impacts of different levels of precipitation severity. This demonstrated capability to support dose-response analysis is a key prerequisite for any formal assessment of network robustness, a core component of resilience. Thus, the IUTF dataset provides the necessary inputs for developing and validating such advanced models.

### Uncovering differentiated macroscopic responses and aggregated impact patterns

**Fig. 9:**
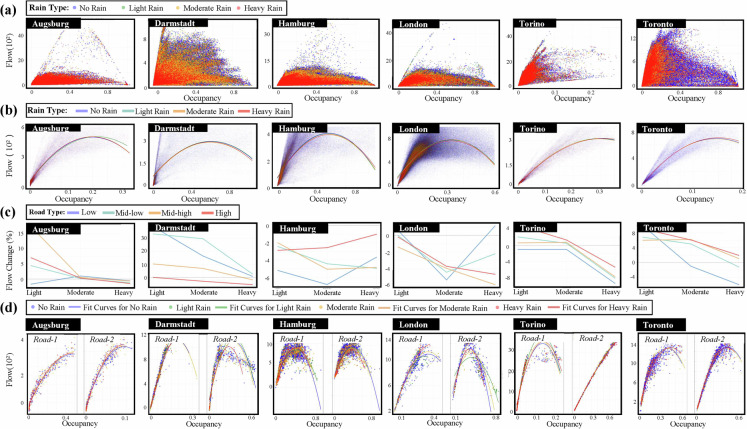
Macroscopic traffic flow characteristics under different rainfall conditions across selected cities. (**a**) Scatter plots of traffic flow versus occupancy for each city, with points coloured by rainfall intensity categories, showing the fundamental traffic flow-occupancy relationship and rainfall impacts, mainly focus on rainfall type, Light, Moderate, Heavy (remove Extreme Rain Type due to the amount of extreme rain events) (**b**) Fitted flow-occupancy curves revealing city-specific variations in traffic capacity and critical density values under different conditions. (**c**) Changes in flow as rainfall intensity increases, demonstrating how precipitation affects fundamental traffic flow parameters. (**d**) Flow-occupancy patterns for high-volume road segments, providing examples of traffic behaviour under different rainfall conditions.

Further validation demonstrated the IUTF dataset’s capacity to reveal how rainfall modifies fundamental macroscopic traffic flow characteristics, a critical aspect for understanding system-level responses. As illustrated in Fig. 9, investigations showed that precipitation events directly perturb core traffic flow-occupancy relationships, leading to observable shifts in traffic capacity and critical density. The dataset’s ability to support such detailed examinations of weather-induced modifications to the fundamental diagram of traffic flow validates its utility for providing the empirical basis needed to develop more nuanced urban resilience strategies.

**Fig. 10:**
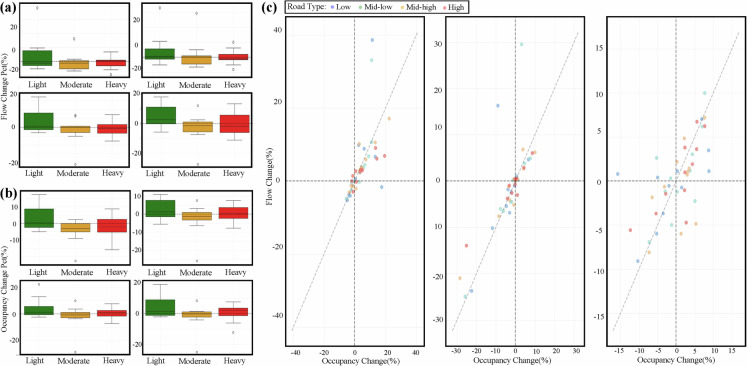
Aggregated analysis of rainfall impact on traffic flow and occupancy across all cities. (**a**) Traffic flow response to rainfall: Box plots showing percentage changes in traffic flow relative to no-rain conditions, categorised by rainfall intensity Light, Moderate, Heavy (remove Extreme Rain Type due to the amount of extreme rain events) and traffic flow levels (Low, Mid-Low, Mid-High, High Flow). (**b**) Occupancy response to rainfall: Box plots showing percentage changes in occupancy relative to no-rain conditions, organised by the same rainfall intensity and traffic flow level categories. (**c**) Coupled flow-occupancy response: Scatter plots of flow change versus occupancy change for each rainfall intensity category, with points coloured by traffic flow levels and fitted with trend lines.

To confirm the broader applicability of such observations, the dataset was used to explore aggregated impact patterns across all 40 cities, as shown in Fig. 10. These analyses consistently revealed systematic changes in both traffic flow and road occupancy attributable to rainfall. The emergence of such distinct and statistically aggregated patterns not only validates the reliability of the observed phenomena but also highlights the IUTF dataset’s strength in facilitating the identification of generalisable insights and systemic vulnerabilities. This capability is vital for informing future evidence-based transportation planning and for building predictive models of network performance degradation under meteorological stress, a key component of resilience.

In summary, the multifaceted technical validation has robustly affirmed the IUTF dataset’s integrity, consistency, and analytical utility. The analyses demonstrated its comprehensive scope, the reliability of its core traffic and precipitation data, and its sensitivity in capturing diverse traffic responses to rainfall—from general diurnal shifts and dynamic peri-event changes to clear dose-response relationships with varying rainfall intensities. Furthermore, the dataset was shown to be capable of revealing complex, heterogeneous impacts on fundamental macroscopic traffic characteristics and statistically significant, aggregated patterns of disruption across diverse urban contexts and road categories. While acknowledging inherent characteristics such as precipitation data resolution and sensor network variability, these comprehensive validation efforts confirm that the IUTF dataset is a valuable and reliable resource, well-suited to support advanced research into urban mobility, flood impacts, and the development of climate adaptation strategies for transportation systems worldwide.

## Usage Note

The IUTF dataset enables a wide range of analytical applications across urban planning, transportation engineering, and climate resilience domains. The IUTF dataset enables a wide range of analytical applications across urban planning, transportation engineering, and climate resilience domains. This section outlines key application areas and provides guidance for researchers conducting resilience-focused studies.

### General applications and data integration

The IUTF dataset enables a wide range of analytical applications across urban planning, transportation engineering, and climate resilience domains. Researchers and practitioners can utilise this dataset for both descriptive and predictive analytical tasks that explore the complex relationships between precipitation events and urban traffic patterns. The dataset supports comprehensive descriptive analyses of how precipitation events influence urban mobility. Researchers can examine the temporal evolution of traffic disruptions before, during, and after rainfall events of varying intensities. The multi-city structure facilitates comparative analyses between different urban environments, revealing how factors such as network topology, infrastructure design, and local policies influence resilience outcomes. These comparisons can identify best practices and transferable strategies for enhancing urban transportation resilience. Beyond descriptive applications, the IUTF dataset enables sophisticated predictive modelling. Transportation planners can develop forecasting tools that incorporate precipitation predictions to anticipate traffic disruptions and optimise management strategies. Climate adaptation researchers can simulate how changing precipitation patterns might affect future urban mobility, informing long-term infrastructure planning and policy development. The dataset’s fine-grained temporal resolution supports the development of early warning systems that can mitigate the impacts of extreme weather events on urban transportation. IUTF data can be seamlessly integrated into existing urban data science workflows with minimal pre-processing. City planners can combine the integrated traffic-precipitation data with socioeconomic indicators to examine how weather-related disruptions differentially impact various urban populations. Emergency management agencies can overlay evacuation route planning with precipitation vulnerability metrics to identify critical intervention points. The dataset’s standardised structure facilitates integration with machine learning frameworks, enabling straightforward implementation of graph-based and sequence-based approaches for complex prediction tasks. The modular and standardised framework developed for the IUTF dataset can be readily extended to incorporate additional meteorological variables. While the current version focuses on precipitation data, the spatio-temporal harmonisation methodology is designed to accommodate other ERA5 variables such as temperature, humidity, wind speed, and atmospheric pressure. Future extensions could also integrate extreme weather event annotations, providing temporal and spatial markers for significant meteorological events that would be particularly valuable for disaster risk studies. The open-source processing framework enables researchers to adapt and extend the dataset according to their specific analytical needs, supporting the development of more comprehensive multi-hazard urban resilience assessments. This extensibility ensures that the IUTF dataset can serve as a foundational platform for increasingly sophisticated climate-transportation interaction studies as new data sources and research questions emerge.

### Quantitative resilience assessment applications

The IUTF dataset provides the essential data components needed to conduct quantitative urban transportation resilience assessments. Researchers can leverage this integrated resource to derive standard resilience metrics and conduct comparative analyses across different urban contexts: **(1) Recovery Curve Analysis**: The dataset’s fine-grained temporal resolution enables the calculation of system recovery curves following precipitation events. Researchers can identify rainfall onset times, track traffic flow changes during and after events, and quantify recovery trajectories. The multi-city structure allows for comparative analysis of recovery patterns, revealing how different urban characteristics (network topology, infrastructure design, management strategies) influence recovery dynamics. Recovery metrics such as recovery time, recovery rate, and residual impact can be systematically computed and compared across the 40 cities. (2) **Robustness Assessment**: The demonstrated dose-response relationship between rainfall intensity and traffic disruption provides the foundation for quantifying system robustness. Researchers can establish performance degradation curves under varying stress levels, calculate robustness indices based on traffic flow reductions, and identify critical rainfall thresholds where system performance significantly degrades. The dataset supports the development of robustness metrics that account for both the magnitude and spatial extent of traffic disruptions. (3) **Adaptive Capacity Evaluation**: The dataset’s integration of traffic flow, occupancy, and network topology data enables investigation of how transportation systems adapt their operational characteristics under stress. Researchers can analyse shifts in fundamental traffic flow relationships, identify adaptive behaviours such as route switching patterns, and quantify the system’s ability to maintain functionality under adverse conditions. The spatial granularity allows for assessment of which network components demonstrate greater adaptive capacity. (4) **Vulnerability Mapping**: By combining precipitation intensity data with traffic impact patterns, researchers can develop detailed vulnerability maps that identify which road segments, network regions, or entire cities are most susceptible to weather-related disruptions. The standardised data structure facilitates the creation of vulnerability indices that can be compared across different urban environments and used to prioritize infrastructure investments. (5) **Multi-Scale Resilience Analysis**: The dataset supports resilience analysis at multiple spatial scales, from individual road segments to entire urban networks. Researchers can investigate how local vulnerabilities aggregate to system-level impacts, examine cascading failure patterns, and assess the effectiveness of different network topologies in maintaining system resilience. The cross-city coverage enables identification of general resilience principles that transcend specific urban contexts.

### Framework extensibility

The spatio-temporal harmonisation framework developed for the IUTF dataset is designed to be modular and transferable. The methodology can be readily adapted to incorporate additional temporal periods, expanded geographical coverage, or supplementary meteorological variables. This extensibility enables researchers to apply the same harmonisation principles to new data sources as they become available, supporting the development of larger-scale or more recent datasets using consistent methodological approaches. The open-source nature of the processing framework facilitates such adaptations and extensions by the research community.

## Data Availability

The IUTF dataset described in this work is publicly available in the Figshare repository at 10.6084/m9.figshare.30022807.v1.

## Code availability

All source code for IUTF data processing is written in Python and hosted under the GNU General Public License 3.0. The package dependencies are listed in a file called requirements.txt. The scripts cover the entire data processing workflow including spatial-temporal harmonisation, data validation, and analysis procedures. The complete processing methodology is explained in a readme markdown file. The code can be found on the GitHub repository(https://github.com/viviRG2024/IUTDF_processing). The repository README provides clear instructions and download links for accessing the complete IUTF dataset.
